# Editorial to the Special Issue “Molecular Aspects in Catalytic Materials for Pollution Elimination and Green Chemistry”

**DOI:** 10.3390/ijms24087033

**Published:** 2023-04-11

**Authors:** Junjiang Zhu

**Affiliations:** Hubei Key Laboratory of Biomass Fibers and Eco-dyeing & Finishing, College of Chemistry and Chemical Engineering, Wuhan Textile University, Wuhan 430200, China; jjzhu@wtu.edu.cn

The Special Issue on “Molecular Aspects in Catalytic Materials for Pollution Elimination and Green Chemistry” encompasses two aims: one is to remove the pollutants produced in the downstream, and the other is to synthesize chemicals by a green route, avoiding the production of pollutants. The aim of this Special Issue is to explore catalysis technology to resolve current environmental problems using photocatalysis, electrocatalysis, and thermocatalysis. This Special Issue also describes research works related to the catalytic removal of pollutants, or the catalytic synthesis of chemicals by a green route.

Xu et al. [1] reported a method of constructing tunable oxygen vacancies (OVs) to accelerate molecular oxygen activation for boosting photocatalytic performance. In this paper, the in situ introduction of OVs on Bi_2_MoO_6_ was accomplished by using a calcination treatment in an H_2_/Ar atmosphere. The introduced OVs not only facilitated carrier separation, but also strengthened the exciton effect, which accelerates singlet oxygen generation through the energy transfer process. Superior carrier separation and abundant singlet oxygen favor photocatalytic NaPCP degradation. The optimal BMO-001-300 sample exhibited the fastest NaPCP degradation rate of 0.033 min^−1^, which is 3.8 times higher than that of the pristine Bi_2_MoO_6_. NaPCP was effectively degraded and mineralized through dechlorination, dihydroxylation, and benzene ring opening. The present work provided a novel insight into ROS-mediated photocatalytic degradation.

Lyulyukin et al. [2] synthesized composite materials based on nanocrystalline anatase TiO_2_ doped with nitrogen and bismuth tungstate by using a hydrothermal method. To determine the correlations between their physicochemical characteristics and photocatalytic activity, the kinetic aspects were studied both in batch and continuous-flow reactors, using ethanol and benzene as test compounds. The researchers found that the Bi_2_WO_6_/TiO_2_-N heterostructure enhanced with Fe species efficiently utilized visible light in the blue region, and exhibited much higher activity in the degradation of ethanol vapor compared to pristine TiO_2_-N. However, the increased activity of Fe/Bi_2_WO_6_/TiO_2_-N had an adverse effect on the degradation of benzene vapor. This result might be due to the fast accumulation of non-volatile intermediates on its surface at high benzene concentrations, which caused a temporary deactivation of the photocatalyst.

Din et al. [3] synthesized three-dimensional (3D) hierarchical Bi_12_O_17_Cl_2_ (BOC) microsphere via a facile solvothermal method by using a binary solvent, which was applied for the photocatalytic degradation of Rhodamine-B (RhB) and Bisphenol-A (BPA). Subsequently, they prepared Co_3_O_4_ nanoparticles (NPs)-decorated BOC (Co_3_O_4_/BOC) heterostructures to further enhance their photocatalytic performances. The BOC microspheres, composed of thin (~20 nm thick) nanosheets, showed a 3D hierarchical morphology and a high surface area. Compared to the BOC, the 20-Co_3_O_4_/BOC heterostructure exhibited enhanced degradation efficiency of RhB (97.4%) and BPA (88.4%). The authors hypothesized that the high surface area, the extension of absorption to visible light region, and the suppression of photoexcited electron-hole recombination are main factors accounting for the better photocatalytic performances. This work provides a new vista to construct high-performance heterostructures for photocatalytic applications.

Phase junctions constructed as photocatalysts show great prospects for organic degradation with visible light. Cai et al. [4] synthesized an elaborate rhombohedral corundum/cubic In_2_O_3_ phase junction (named MIO) combined with polymeric carbon nitride (PCN) by using an in situ calcination method. The authors attributed the excellent performance of MIO/PCN to the intimate interface contact between MIO and PCN, which provides a reliable charge transmission channel, thereby improving the separation efficiency of charge carriers. This work illustrates that MOF-modified materials have a great potential for solving environmental pollution without generating secondary pollution.

The need for effective and rapid processes for photocatalytic degradation has led to an increased interest in finding more sustainable catalysts for antibiotic degradation. Bai et al. [5] provided an overview on the removal of pharmaceutical antibiotics through photocatalysis, and the recent progress using different nanostructure-based photocatalysts. They also reviewed the possible sources of antibiotic pollutants released through the ecological chain, and the consequences and damages caused by antibiotics in wastewater on the environment and human health. The fundamental dynamic processes of nanomaterials and the degradation mechanisms of antibiotics were discussed, and recent studies regarding different photocatalytic materials for the degradation of some typical and commonly used antibiotics were summarized. Finally, the challenges and future opportunities for photocatalytic degradation of commonly used antibiotics were highlighted.

Formate is a desirable product of an electrochemical CO_2_ reduction (CO_2_RR) and has great economic value. Li et al. [6] reported a facile one-step method to synthesize nitrogen-doped bismuth nanosheets (N-BiNSs), as electrocatalysts, for CO_2_RR to produce formate. The N-BiNSs exhibited a high formate Faradic efficiency with a stable current density. Moreover, the N-BiNSs for CO_2_RR yielded a large current density for formate production in a flow-cell measurement, achieving the commercial requirement. The authors found that nitrogen doping could induce charge transfer from the N atom to the Bi atom, thus modulating the electronic structure of N-Bi nanosheets. Density Functional Theory results demonstrated that the N-BiNSs reduced the adsorption energy of the *OCHO intermediate and promoted the mass transfer of charges. This study provides a valuable strategy to enhance the catalytic performance of bismuth-based catalysts for CO_2_RR by using a nitrogen-doping strategy.

Long et al. [7] synthesized a nanometer-size Co-ZIF (zeolitic imidazolate frameworks) catalyst for selective oxidation of toluene to benzaldehyde under mild conditions, which showed a BET surface area of 924.25 m^2^/g. The authors investigated the effect of reaction temperatures, oxygen pressure, mass amount of N-hydroxyphthalimide (NHPI), and reaction time on the catalytic activity. The results showed that the Co-ZIF catalyst gave the best result of 92.30% toluene conversion and 91.31% selectivity to benzaldehyde under 0.12 MPa and 313 K. This new nanometer-size Co-ZIF catalyst exhibits good application prospects in the selective oxidation of toluene to benzaldehyde.

Zhang et al. [8] prepared the PtSn catalysts for propane dehydrogenation by incipient-wetness impregnation using a dendritic mesoporous silica support. The authors found that changing the Pt/Sn ratios influenced the interaction between Pt and Sn. The best catalytic performance was obtained from the Pt_1_Sn_2_/DMSN catalyst, with an initial propane conversion of 34.9%. They proposed that the good catalytic performance of this catalyst is a result of the small nanoparticle size of PtSn and the favorable chemical state and dispersion degree of Pt and Sn species.

The efficient recycling of valuable resources from rolling oil sludge (ROS) is meaningful. Gao et al. [9] reported the recycling of solid Fe resources from ROS by a catalytic hydrogenation technique, and discussed its catalytic performance for CO oxidation. The solid Fe resources calcinated in air (Fe_2_O_3_-H) exhibited comparable activity for CO oxidation to those prepared by the calcinations of ferric nitrate (Fe_2_O_3_-C). Following this, the authors supported the Fe resources on 13X zeolite, and pretreated the sample with CO atmosphere, which led to a complete CO conversion at 250 °C on the 20 wt.% Fe_2_O_3_-H/13X sample.

Graphitic carbon nitride (g-C_3_N_4_) is thoroughly studied owing to its remarkable properties exhibited in photocatalysis. Lin et al. [10] summarized the research progress on the synthesis of g-C_3_N_4_ and its coupling with single- or multiple-metal oxides, and its photocatalytic applications in energy production and environmental protection, including the splitting of water to hydrogen, the reduction of CO_2_ to valuable fuels, the degradation of organic pollutants, and the disinfection of bacteria. At the end, challenges and prospects in the synthesis and photocatalytic applications of g-C_3_N_4_-based composites were proposed, and an outlook was provided.
